# Beyond Traditional Newspaper Advertisement: Leveraging Facebook-Targeted Advertisement to Recruit Long-Term Smokers for Research

**DOI:** 10.2196/jmir.5502

**Published:** 2016-06-15

**Authors:** Lisa Carter-Harris, Rebecca Bartlett Ellis, Adam Warrick, Susan Rawl

**Affiliations:** ^1^ Indiana University School of Nursing Indianapolis, IN United States; ^2^ Indiana University Bloomington Bloomington, IN United States

**Keywords:** facebook, recruitment methods, smokers, older

## Abstract

**Background:**

Smokers are a stigmatized population, but an important population to reach for the purpose of research. Therefore, innovative recruitment methods are needed that are both cost-effective and efficacious in recruiting this population.

**Objective:**

The aim of the present article was to evaluate the feasibility of Facebook-targeted advertisement to recruit long-term smokers eligible for lung cancer screening for a descriptive, cross-sectional survey.

**Methods:**

A social media recruitment campaign was launched using Facebook-targeted advertisement to target age and keywords related to tobacco smoking in the Facebook users profile, interests, and likes. A 3-day newspaper advertisement recruitment campaign was used as a comparison. The study that used both recruitment methods aimed to test the psychometric properties of 4 newly developed lung cancer screening health belief scales. Data were collected via cross-sectional survey methodology using an Web-based survey platform.

**Results:**

The Facebook-targeted advertisements were viewed 56,621 times over an 18-day campaign in 2015 in the United States. The advertisement campaign yielded 1121 unique clicks to the Web-based survey platform at a cost of $1.51 per completed survey. Of those who clicked through to the study survey platform, 423 (37.7%) consented to participate; 92 (8.2%) dropped out during completion of the survey yielding a final study pool of 331 completed surveys. Recruitment by newspaper advertisement yielded a total of 30 participants in response to a 3-day advertisement campaign; recruitment efficacy resulted in 10 participants/day at $40.80 per completed survey. Participants represented current (n=182; 51%) and former smokers (n=178; 49%) with a mean age of 63.4 years (SD 6.0). Cost of the advertisement campaign was $500 total for the 18-day campaign.

**Conclusions:**

Recruitment by Facebook was more efficacious and cost-effective compared with newspaper advertisement. Facebook offers a new venue for recruitment into research studies that offer the potential for wider reach at a lower cost while providing privacy and flexibility for potential study participants. The study’s findings extend recent work of other researchers who have demonstrated Facebook’s utility with younger smokers, and Facebook is an effective tool to recruit older smokers. Furthermore, Facebook is a cost-effective alternative to traditional newspaper advertisement offering a new, affordable venue to recruit large numbers of older smokers efficiently.

## Introduction

Lung cancer is the deadliest cancer-related diagnosis worldwide regardless of gender or ethnicity with most patients diagnosed at an advanced stage [1]. For the first time, there is a screening test for high-risk individuals (*defined as current or former smokers who have quit within the past 15 years who have a 30 pack-year tobacco smoking history*) [2,3]. Lung cancer screening with low-dose computed tomography (LDCT) in long-term smokers has been shown to decrease relative lung cancer-related mortality by 20% [2]. An LDCT is a newer form of a computed tomography scan that uses lower doses of radiation to take a series of 3D radiographs of the lungs. These images are detailed and can show early-stage lung cancers that may be too small for conventional chest radiographs to detect [2]. In response to empirical findings from the National Lung Screening Trial, the US Preventive Services Task Force issued lung cancer screening guidelines recommending annual LDCT of the chest for high-risk individuals in 2013 [4].

As lung cancer screening becomes more widely implemented, participation is likely to be influenced by many factors, including individual-, provider-, and health care system-related variables. To determine factors that influence lung cancer screening participation, understanding perspectives of individuals eligible for screening is essential. Current lung cancer screening guidelines target long-term current and former smokers [3]. However, recruitment of smokers can be a challenge, and such research can be limited by the ability to access this target population. Smoking-related stigma has been implicated in timing of medical help-seeking behavior in symptomatic individuals later diagnosed with lung cancer [5] and quality of life in current and former smokers diagnosed with lung cancer [6]. Smoking-related stigma may serve as a barrier to recruitment of this important population. Current and former smokers may worry about being blamed for their smoking history as well as feel like social outcasts for smoking, increasing a sense of internalized stigma [7,8]. They may also fear having to endure a lecture from their health care provider about their current smoking status. For researchers wishing to recruit smokers, traditional methods such as face-to-face recruitment and fliers placed in high-traffic areas may not be as successful as recruitment targeting other demographics for research studies [9]. In addition, newspaper advertisement may be costly. Facebook is a relatively new venue for recruitment into research studies and offers the potential for wider reach at a lower cost while providing privacy and flexibility for potential study participants. Innovative methods are needed that are both cost-effective and efficient in recruiting this potentially hard-to-recruit population.

Facebook has previously been established as a viable option to recruit young adults into health-related research [10-17] and may be a successful recruitment tool for older adults. Facebook has been a successful recruitment tool to reach adolescents and young adults for a range of study purposes including exploring mental health issues [13,18], examining pubescent hormonal effects in early adolescence [19], and recruiting for a variety of Web-based intervention studies [18,20,21]. Facebook may also be a beneficial resource for retention in longitudinal studies as previous studies have demonstrated its utility in retaining adolescents via social media [14-15]. Facebook has also been a successful recruitment strategy for reaching “hard-to-reach” populations such as men who have sex with men [22] and exploring taboo topics with adolescents and young adults such as abortion [16]. Finally, studies targeting young adult smokers have demonstrated Facebook advertisements as a successful recruitment tool [9,17,23,24]. Therefore, our study sought to determine whether Facebook would be a successful recruitment tool in a new domain: older, long-term smokers.

As of March 2015, Facebook reported 936 million active daily users worldwide [25]. In the United States, 71% of adults use Facebook [26]. Facebook has also grown in use among older individuals and is visited by 63% of individuals aged 50 to 64 years and by 56% of individuals aged 65 years and older in a typical day [26]. Facebook offers the ability for the researcher to market a recruitment campaign of advertisements targeted by age, location, and keywords identified in the profile or interest list of potential participants. This strategy of targeted advertisement has the potential to engage current and former smokers as research participants while maintaining a sense of privacy for the individual potentially decreasing the perception of associated stigma. The purpose of this article was to describe the method by which a national sample of older long-term current and former smokers was successfully recruited into a descriptive survey study using Facebook-targeted advertisement.

## Methods

### Sample

This Facebook sample was recruited as part of a larger overall study to psychometrically test 4 newly developed scales to measure health beliefs about lung cancer screening. The findings of the psychometric study are published [27]. For the larger study, we aimed to recruit men and women who were eligible for lung cancer screening and included individuals between the ages of 55 and 77 years who were current or former smokers who had quit within the past 15 years and had a 30 pack-year tobacco smoking history. Pack-year is defined as the number of packs of cigarettes smoked per day multiplied by the number of years smoked. Individuals diagnosed with lung cancer were excluded from the study. It should be noted that the age range for the inclusion criteria of the psychometric study was 55 to 77 years. However, Facebook-targeted advertisement does not currently offer the ability to narrow age range in the 65-years-and-older category. Therefore, the age targeted for the purposes of the Facebook advertisement was 55 years and older with the ability to analyze Facebook advertisement metrics by 55 to 64 years and 65 years and older as discrete ranges. Two recruitment strategies were used: (1) a Facebook advertisement campaign and (2) a newspaper advertisement. The required sample size, based on the planned statistical analyses to evaluate the psychometric properties of the lung cancer screening health belief scales, was 300 completed surveys. Institutional review board approval was obtained from Indiana University before recruitment of study participants.

### Procedures

#### Facebook Advertisement Campaign

A social media recruitment campaign was launched using Facebook-targeted advertisement over an 18-day period. Facebook uses 2 types of advertisements that can appear in different locations on the screen depending on platform used to access Facebook (ie, desktop or mobile app). To best understand these advertisement locations, key terminology specific to Facebook advertisement must be explained to include: (1) newsfeed; (2) impressions; (3) reach; and (4) clicks to website. A *newsfeed* is a constantly updating list of stories in the middle of the Facebook user’s homepage. The newsfeed can include status updates, photos, videos, links, application activity, and likes from people, pages, and groups followed by the Facebook user. Advertisements also appear in a Facebook user’s newsfeed. The 2 types of advertisements specific to the desktop platform include: (1) standard desktop advertisements that appear in the right hand column next to the newsfeed on the Facebook user’s homepage and (2) newsfeed advertisements that appear in the constantly updating newsfeed located in the middle of the Facebook user’s homepage. For mobile app users, the newsfeed advertisement appears only in the middle of the constantly updating newsfeed. An *impression* refers to the number of times the advertisement entered the screen for the first time (ie, is served to someone) either in their desktop newsfeed, mobile newsfeed, or as a right hand column advertisement. *Reach* refers to the number of people to whom the advertisement was shown. A *click to website* refers to a unique Facebook user clicking the weblink embedded in the Facebook advertisement that is redirected to the advertised website. Another method to reach potential study participants in Facebook is to target the Facebook advertisement to the audience network of a Facebook group page. An *audience network* refers to individuals who have liked a Facebook group page. In the case of this study, the researchers set up a Facebook group page called Healthy Lungs Initiative with the purpose of providing general lung health information to recruit individuals interested in lung health issues. The Healthy Lungs Initiative Facebook group page was set up at the start of the study and remains active.

**Figure 1:**
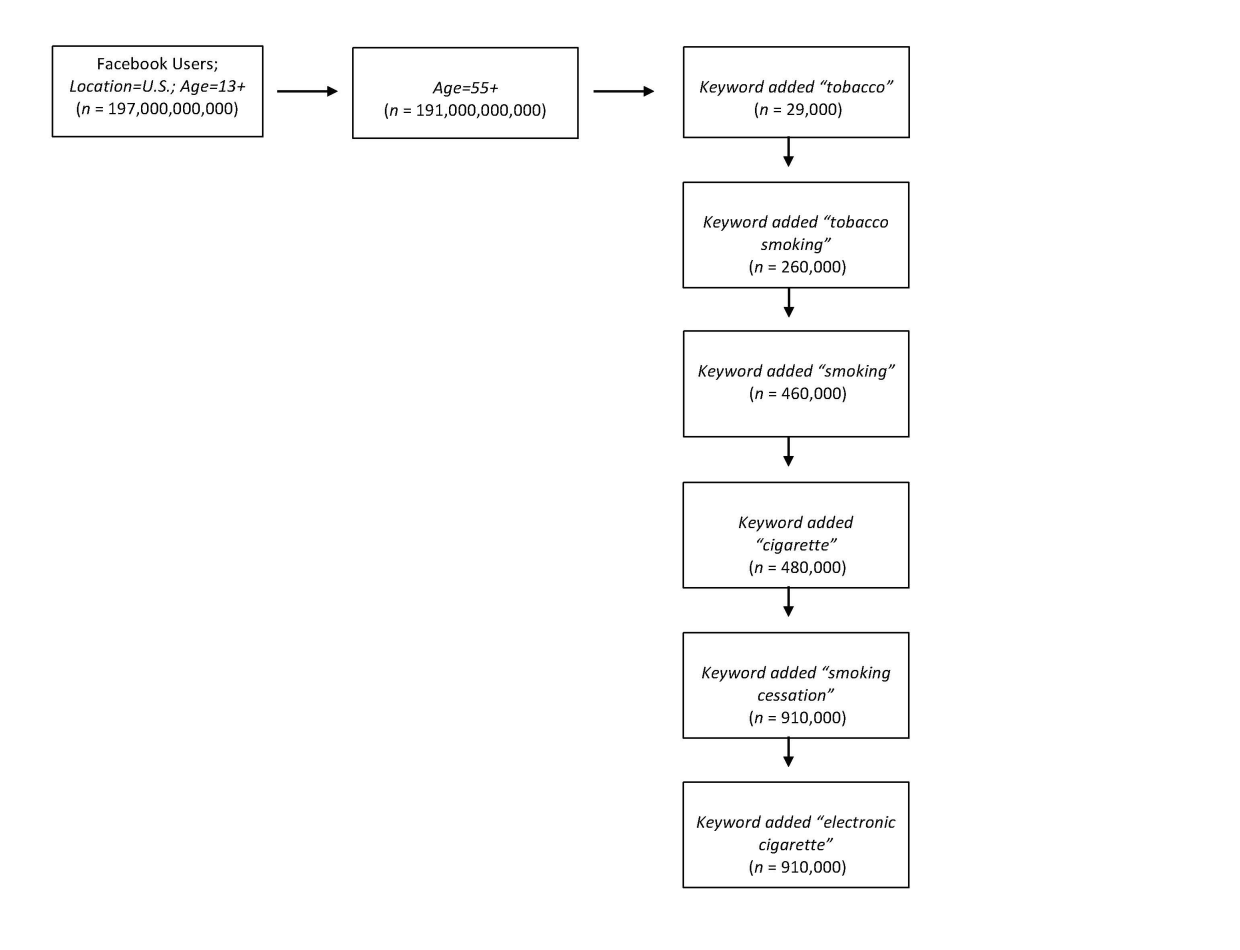
Keyword Metrics of Potential Reach for Facebook Targeted Advertisement.

**Figure 2:**
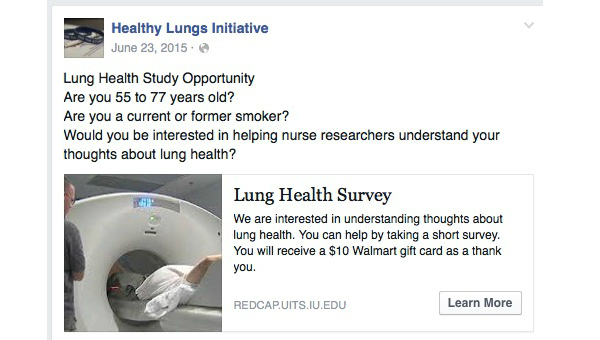
Facebook Recruitment Advertisement.

A recruitment advertisement targeting Facebook users whose age were 55 years and older and lived in the United States was used. Keywords used in targeting the selected group for our recruitment included “tobacco,” “tobacco smoking,” “smoking,” “smoking cessation,” “cigarettes,” and “electronic cigarettes” using both standard desktop and newsfeed advertisements (mobile and desktop). When we defined our audience during creation of the advertisement using these keywords, age, and location, we had a potential reach of 910,000 people. Please see Figure 1 for metrics related to potential reach specific to each stage of the keyword targeting process. Standard desktop and newsfeed advertisements included a short headline, a picture of an individual getting an LDCT scan for lung cancer screening, a short description of the study, and a link to the study’s Web-based survey (see Figure 2). The study’s Web-based survey was conducted through an external website using the Research Electronic Data Capture (REDCap) software system. REDCap is a secure Web-based application for building and managing Web-based surveys and databases. As previously mentioned, the advertisement was connected to a Facebook group page set up specifically for this study by the researchers called Healthy Lungs Initiative. The advertisement was reviewed and approved by Facebook staff before being released per Facebook policy. Dates for the recruitment campaign were specified, and a lifetime spending limit for the recruitment campaign was set at $500 during the creation of the advertisement. In addition, we followed procedures to reduce participant misrepresentation as described by Kramer et al [28]. These include (1) prohibiting open access to the survey platform by embedding a weblink that redirects to a screening survey; (2) requiring screening questions to screen out individuals who do not meet the inclusion criteria; (3) incorporating a survey time stamp to examine initiation of survey versus survey completion time span; and (4) identifying item pairs that should be consistent.

#### Newspaper Advertisement

A newspaper advertisement was placed in *The Indianapolis Star* newspaper in Indianapolis. Indiana is ranked 6th highest in adult smoking rates nationally with 21.9% of the state’s adult men and women self-reporting as current smokers [1]. The advertisement was a 3.24” x 5” black and white announcement that ran for 3 consecutive days in the lifestyle section of the newspaper. The advertisement featured a black and white picture of diverse older adults of both genders and details related to eligibility criteria, what the study involved, and information to participate. The following 3 ways to participate were included in the advertisement: (1) visit a weblink to access the Web-based version of the survey; (2) email or call the research office to request a mailed copy of the survey; or (3) call the research office to complete the survey by telephone.

## Results

The primary objective of this study was to evaluate the feasibility of Facebook-targeted advertisement to recruit long-term smokers eligible for lung cancer screening for a descriptive, cross-sectional survey. Although not a direct comparison in length of advertisement and geographic location, we present the newspaper advertisement results as a comparison of cost, time, and number of participants recruited per day.

### Participant Characteristics

Of the 361 participants, the majority were recruited by Facebook advertisement (92%; n=331). Across both the recruitment methods, the majority were female (58%; n=211), non-Hispanic Caucasian (91%; n=327), high school graduates or higher (96%; n *=* 347), and equally representative of current (51%; n=182) and former smokers (49%; n=177). The mean age was 63.4 years (SD 6.0). Please see Table 1 for a complete list of participant sociodemographic characteristics in total and by recruitment method.

**Table 1:** Participant sociodemographic characteristics by recruitment method (N=361).

		Total (N=361)^b^	Newspaper^b^(n=30)	Facebook^b^(n=331)
**Gender**, n (%)				
	Female	211 (58)	17 (57)	194 (58)
	Male	150 (42)	13 (43)	137 (41)
**Race or ethnicity**, n (%)				
	Caucasian	327 (91)	22 (73)	305 (92)
	African-American	28 (8)	8 (27)	20 (6)
**Education**, n (%)				
	<High school	15 (4)	1 (3)	14 (4)
	High school graduate	104 (29)	6 (20)	98 (30)
	Some college	154 (43)	15 (50)	139 (42)
	College graduate	15 (4)	8 (27)	81 (24)
**Annual income**, n (%)				
	Less than $25,000	116 (32)	12 (40)	104 (31)
	$25,000-50,000	168 (47)	11 (37)	157 (47)
	More than $50,000	74 (21)	6 (20)	68 (21)
**Insurance status**, n (%)				
	Government sponsored	217 (60)	24 (80)	193 (58)
	Private insurance	132 (37)	5 (17)	127 (38)
	No insurance	13 (3)	1 (3)	12 (4)
**Smoking status**, n (%)				
	Current smoker	182 (51)	20 (67)	162 (49)
	Former smoker	177 (49)	10 (33)	167 (51)
**Family history of lung cancer**, n (%)				
	Yes	63 (18)	4 (13)	59 (18)
	No	299 (82)	26 (87)	273 (82)
**Age (years), mean (SD)** ^a^		63.4 (6)	63.6 (6)	63.1 (6)

^*a*^SD: standard deviation.

^b^ *Note.* Column percentages may not add to 100% due to missing data.

### Newspaper Advertisement

One black and white newspaper advertisement ran over 3 consecutive days (Monday, Tuesday, and Wednesday) at a total cost of $1224. Subscribers aged 55 years and older (N=230,742) received the newspaper those 3 days. Therefore, the newspaper advertisement had a potential reach of 230,742 individuals aged 55 years and older who potentially saw the advertisement over the 3-day campaign. A total of 42 individuals responded to the advertisement; among them, 30 participants met the inclusion criteria, agreed to participate in the study, and completed the survey. Among the 30 participants, 21 (70%) completed the survey via the Web, 4 (13%) completed by telephone, and 5 (17%) completed a paper copy of the survey by email. Recruitment over the 3-day newspaper advertisement campaign averaged 10 participants per day (30 total participants or 3-day campaign=10), and cost of recruitment by newspaper advertisement was $40.80 per completed survey ($1224/30=$40.80).

### Facebook Advertisement Respondents

**Figure 3:**
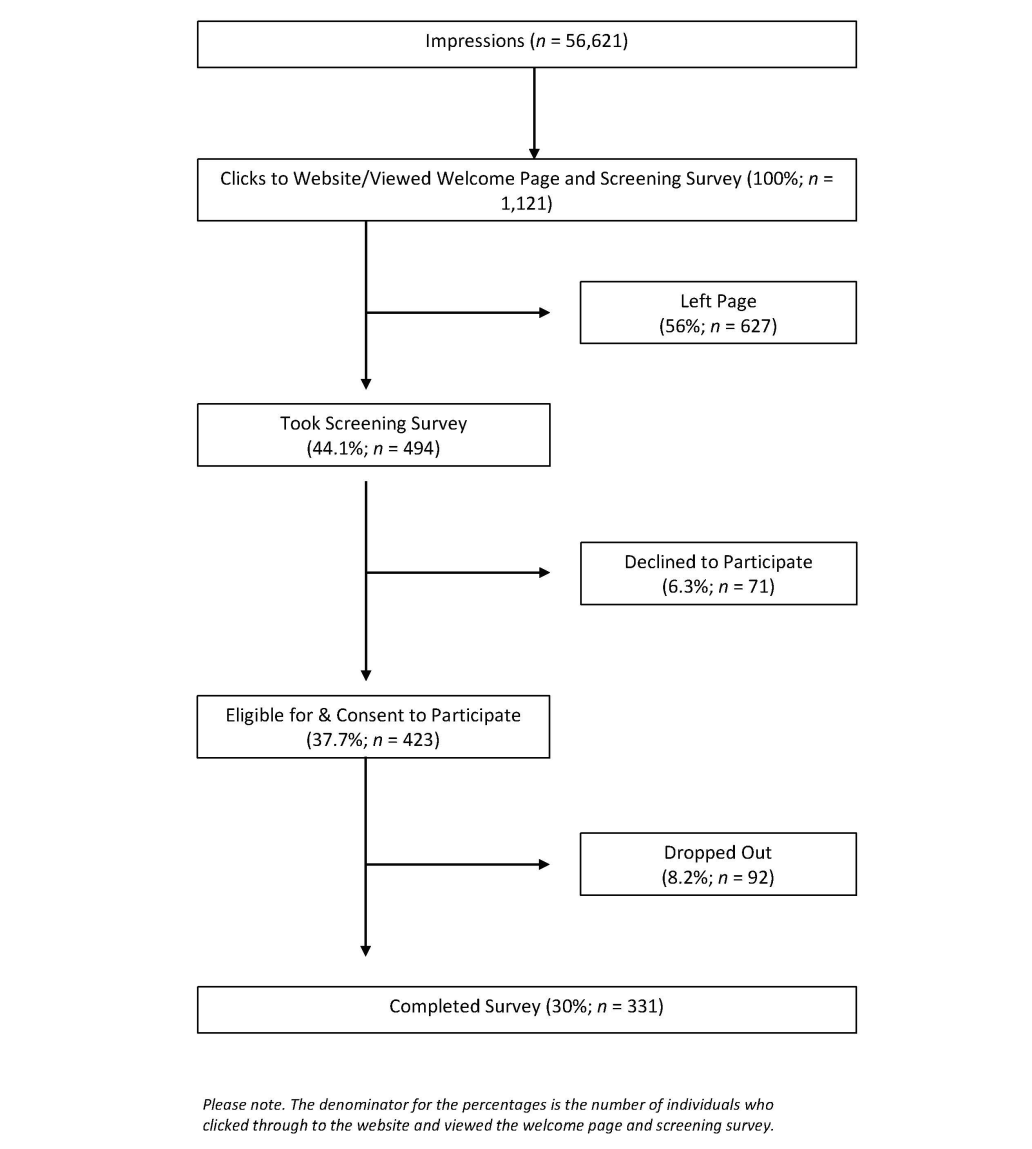
Flow diagram of clicks to website, eligibility, consent to participate, and dropouts.

During the 18-day Facebook recruitment campaign, 1121 unique Facebook users viewed, clicked on the advertisement, and were directed to the survey welcome page and screening survey. As depicted in the flow diagram in Figure 3 , of the 1121 people who viewed the survey welcome page and screening survey, 423 (37.7%) agreed to participate, but 92 (8.2%) people dropped out during the course of the survey. Although the survey was designed with the option to leave any item blank after proceeding past the screening survey, there were less than 5% missing data for all survey items. Recruitment by Facebook advertisement yielded 331 participants enrolled into the study.

### Facebook Recruitment Campaign Results

**Figure 4:**
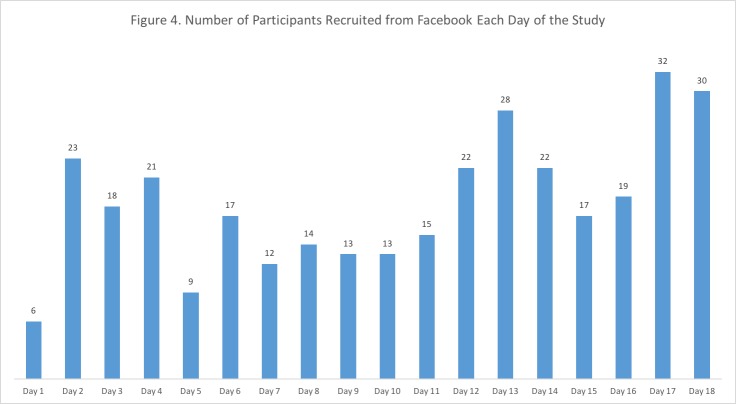
Number of Participants Recruited from Facebook Each Day of the Study.

Over the 18-day Facebook recruitment campaign, the advertisement made 56,621 impressions yielding 1121 unique clicks to our Web-based survey at an overall cost of $500. Advertisements appeared on the right side of the desktop screen, within the desktop newsfeed as the potential participant scrolled, within the mobile newsfeed, or in the audience network of individuals who liked the Healthy Lungs Initiative Facebook group page. Newsfeed advertisements on a mobile device resulted in more unique users with 42,059 impressions and 894 unique clicks to the Web-based survey site than the other advertisement placements (see Table 2 for a complete list of Facebook-advertising metrics). In addition, 7 unique clicks were generated from the Healthy Lungs Initiative Facebook group page. As mentioned previously, the Facebook group page was created, in conjunction with the Facebook-targeted advertisement for this study, as a platform for messages about general lung health issues. During the 18-day Facebook advertisement period, the Facebook group page obtained 29 likes. Recruitment over the 18-day Facebook advertisement campaign averaged 18 participants per day, and cost of recruitment was $1.51 per completed survey ($500/331=$1.51). See Figure 4 for number of participants recruited by Facebook advertisement per day.

**Table 2:** Characteristics of an 18-day Facebook-targeted advertisement campaign with Facebook-advertising metrics (N=331).

Variable		Reach	Unique Clicks to Website	Spent (cost per click)
**Total Facebook users and budget**		56,621	1121	$500
**Advertisement placement**				
	Desktop right side	4527	22	$0.32
	Desktop newsfeed	7409	190	$0.30
	Mobile newsfeed	42,059	894	$0.48
	Audience network^a^	1015	7	$0.11
**Gender**				
	Women	35,581	722	$0.48
	Men	20,269	378	$0.40
	Unspecified	772	21	$0.29
**Age, years**				
	55-64	31,004	602	$0.44
	65+	25,617	519	$0.46

^a^Audience network refers to individuals who saw advertisement on Facebook page (Healthy Lungs Initiative).

## Discussion

Long-term current and former smokers can be challenging to reach and effectively recruit into research studies possibly secondary to perceived smoking-related stigma. However, this study demonstrated that Facebook is a viable recruitment method for this population. Facebook advertisement was more effective in cost and number of participants recruited compared with newspaper advertisement. Facebook advertisement cost was $1.51 per completed survey and averaged 18 recruited eligible participants per day compared with $40.80 per completed survey and an average of 10 recruited eligible participants per day with newspaper advertisement. In general, Facebook advertisement had minimal resource use, including time and cost related to research personnel. Management of the Facebook advertisement campaign involved less than 1 hour per day spent monitoring enrollment and stability of the embedded weblink to the survey. The newspaper advertisement, however, required greater personnel and time investment to answer telephone calls, respond to voice mail messages, and administer the survey by telephone for participants who chose this option.

In addition to the low cost benefit, leveraging Facebook’s ability to target specific study populations by demographic characteristics, location, and keywords in the Facebook users’ profile, and interest list is of value to the researcher. This ability offers the opportunity to target recruitment advertisements based on multiple combinations of population characteristics important to their study. For this study, targeting by location, age, and keywords related to tobacco smoking and smoking cessation provided a potential reach of 910,000 unique Facebook users. We found this approach to be helpful in reaching our target audience of long-term current and former smokers because the keywords were likely personally relevant for the target population needed for the study. Targeting Facebook advertisements using personally relevant keywords also has the potential to decrease advertisements being shown to individuals who are not in the intended target population. Facebook also offers the opportunity to reach older individuals via their mobile device. In this study, most Facebook advertisements were seen in an individuals’ mobile newsfeed (74.3%; n=42,059) indicating that many individuals aged 55 years and older are accessing Web-based content using their smartphones or tablet devices. Although Facebook advertising metrics did not allow for a breakdown by age and device type, this is a new trend that should be considered further for potential recruitment opportunities with this demographic and studied.

Facebook-targeted advertisement has great potential as a recruitment strategy for other studies. Previous studies using Facebook to recruit research participants have used a Facebook study group page to first recruit Facebook users to “like” and join the group page followed by direct advertisement to page group members via postings on the group page [29,30]. Our findings extend the work of Valdez et al [29] who assessed leveraging Facebook’s social structure for research recruitment and concluded that Facebook recruitment was feasible for recruiting small samples for qualitative research but not for recruiting larger samples for quantitative research. Both recruitment strategies in the study by Valdez et al depended on an intermediary within the social structure of Facebook to recruit participants. For the first method tested, the study by Valdez et al created a study-related Facebook group page and asked administrators of other Facebook group pages to publicize the Valdez study-related Facebook group page to their members. The quantitative survey was then advertised on the study-related Facebook group page. The second recruitment method tested involved obtaining consent of other established Facebook group pages and posting a link to the study’s survey on those pages. Our study did not use an intermediary component as the primary mode of recruitment, rather, we targeted keywords of individual profile “likes” and “interests” that the researchers felt were likely to appear in the profile of long-term current and former smokers (examples include “tobacco,” “tobacco smoking,” “smoking,” “smoking cessation,” “cigarettes,” and “electronic cigarettes”). Most importantly, our findings extend the work of researchers who have demonstrated Facebook’s utility with younger smokers [9,17,23,24] as we demonstrate Facebook is an effective recruitment tool for older smokers. Furthermore, our findings extend the work of Frandsen et al [12] by evaluating the yield of the recruitment strategy by monitoring the time (in days) that the Facebook-targeted advertisement was in use, and clicks to website generated and sociodemographic characteristics of the participants recruited. In addition, leveraging creation of a Facebook group page that may appeal to the intended audience may be of benefit to recruitment efforts. To date, the Healthy Lungs Initiative Facebook group page has 114 likes in the 10 months since set up with very minimal effort. With a more concerted effort such as daily or weekly posting of general information to engage the Facebook group page audience, the audience network has the potential to grow. By attracting an audience that may have similar interests as a researcher’s target population, this venue within Facebook offers the potential for longer-term participant engagement for future research opportunities.

### Limitations and Unique Considerations

As with all studies, this study is not without limitations. Although Facebook recruited more participants at a lower cost, it is important to note that the study design is limited by inequitable comparison of recruitment methods. The Facebook advertisement was a nationwide recruitment campaign, whereas cost limited the study’s newspaper advertisements to 1 high-readership newspaper in the Midwest United States. Although participant sociodemographic characteristics were largely similar, recruitment efficacy comparisons may be impacted. Future efforts to evaluate recruitment efficacy by Facebook versus newspaper advertisement should be designed to compare recruitment in the same geographic location. It is also important to note that although the newspaper advertisement had a 3-day reach of 230,742 individuals aged 55 years and older, it is highly probable that not all 230,742 individuals saw the advertisement given the nature of print media. Similarly, the 56,621 Facebook users who were likely exposed to the Facebook advertisement in their newsfeed have a probability that not all will have paid attention to and read the advertisement. However, Facebook advertisements requires an individual to click the advertisement when seen to take advantage of the opportunity being presented as opposed to print media, which can be read and returned to later to take advantage of the opportunity presented in the advertisement. As researchers design future recruitment strategies, it is important to consider how a recruitment advertisement is presented and consumed to maximize potential reach.

Participants recruited via Facebook were primarily Caucasian indicating that Facebook may not be the best recruitment method for older long-term current and former smokers from racially and ethnically diverse backgrounds. This potential limitation of sample diversity necessitates comparing the study outcomes by groups to ensure there are no significant differences. Therefore, the potential lack of representativeness of the study sample may be an important limitation of Facebook advertisement for recruitment. Future research involving lung cancer screening–eligible current and former smokers using Facebook-targeted advertisement as a recruitment method should monitor recruitment numbers of African-Americans and other racially and ethnically diverse groups closely. Although the sample recruited was predominately non-Hispanic Caucasian men and women, Facebook’s advertising program does offer the ability to target advertisement campaigns by zip code. Future studies could be designed to target ethnically diverse zip codes throughout a particular location to increase the numbers of participants from underrepresented populations. In addition, it is also important to note that the success of recruitment in this study may be directly related to its low burden (ie, 1-time survey directly accessed by a weblink with a $10 gift card incentive).

Finally, Facebook advertisement has the potential to recruit long-term smokers who may not be interested in responding to recruitment advertisements in high-traffic, highly visible areas or in methods that require the potential participant to contact the researcher directly. Facebook allows a degree of anonymity not present in these other forms of research recruitment, which may be successful with individuals who may have concerns related to stigma. However, it should be noted that Facebook could also enhance feelings of stigma because of a constant reminder of their smoking status being presented via a Facebook advertisement recruiting smokers. Future research is needed to explore potential differences in stigma levels in those screening-eligible smokers recruited via Facebook versus other methods.

### Conclusions

Facebook-targeted advertisement offers the opportunity to reach a large number of potential study participants at a low cost while providing privacy and anonymity increasing the likelihood of recruiting a population that may experience stigma. Health behavior research related to lung cancer screening participation is important, and recruitment of current and former long-term smokers is critical to the success of this research. Smokers may experience perceived stigma, which may influence the success of recruitment by more traditional methods. Researchers who desire to reach individuals engaging in stigmatized behaviors (such as smoking) should consider Facebook as a viable option for study recruitment.

In addition, traditional recruitment strategies such as newspaper advertisement can be costly from a financial and personnel perspective. Recruitment of older current and former long-term smokers via Facebook-targeted advertisement offers a novel, efficient, efficacious, and cost-effective recruitment strategy that was successful in this cross-sectional, descriptive study.

## Abbreviations

LDCT: low-dose computed tomography
REDCap: Research Electronic Data Capture
